# Polythia­zide

**DOI:** 10.1107/S1600536810022105

**Published:** 2010-06-16

**Authors:** Thomas Gelbrich, Mairi F. Haddow, Ulrich J. Griesser

**Affiliations:** a Institute of Pharmacy, University of Innsbruck, Innrain 52, 6020 Innsbruck, Austria

## Abstract

The crystal structure of the title compound, C_11_H_13_ClF_3_N_3_O_4_S_3_ (systematic name: 6-chloro-2-methyl-3-{[(2,2,2-trifluoro­eth­yl)sulfan­yl]meth­yl}-3,4-dihydro-2*H*-1,2,4-benzothia­diazine-7-sul­f­on­amide 1,1-diox­ide; CRN: 346–18–9), exhibits a two-dimensional network of hydrogen-bonded mol­ecules parallel to (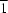
01). The NH and NH_2_ groups act as donor sites and the sulfonyl O atoms as acceptor sites in N—H⋯O hydrogen bonds, and a C—H⋯O interaction also occurs. The thiadiazine ring adopts an envelope conformation with the N atom bonded to sulfur at the tip of the flap, and the methyl substituent is in an axial position.

## Related literature

For the preparation of polythia­zide, see: McManus (1961 ▶). For a comprehensive description of polythia­zide, see: Negendra Vara *et al.* (1991 ▶). For a preliminary crystallographic study at room temperature, see Dupont & Dideberg (1970 ▶). For crystal structures of polymorphs and solvates of related thia­zide compounds, see: Zhou *et al.* (2006 ▶); Johnston *et al.* (2007*a* ▶,*b* ▶); Johnston *et al.* (2007 ▶); Fernandes, Florence *et al.* (2006 ▶); Fernandes, Shankland *et al.* (2007 ▶); Johnston *et al.* (2008 ▶); Fabbiani *et al.* (2007 ▶); Fernandes, Johnston *et al.* (2007 ▶); Fernandes, Leech *et al.* (2007 ▶).
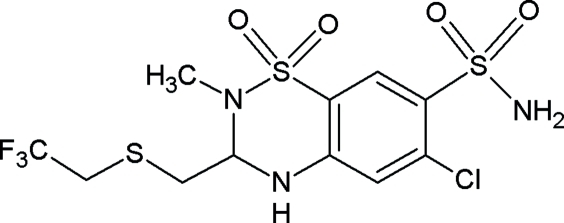


## Experimental

### 

#### Crystal data

C_11_H_13_ClF_3_N_3_O_4_S_3_
*M* *_r_* = 439.87
Monoclinic, 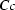

*a* = 14.6659 (7) Å
*b* = 9.5498 (6) Å
*c* = 13.6720 (7) Å
β = 116.149 (3)°
*V* = 1718.87 (16) Å^3^
*Z* = 4
Mo *K*α radiation
μ = 0.64 mm^−1^
*T* = 120 K
0.12 × 0.10 × 0.06 mm

#### Data collection

Bruker-Nonius Roper CCD camera on κ-goniostat diffractometer
Absorption correction: multi-scan (*SADABS*; Sheldrick, 2007 ▶) *T*_min_ = 0.927, *T*_max_ = 0.963
9021 measured reflections
3197 independent reflections
2768 reflections with *I* > 2σ(*I*)
*R*_int_ = 0.055

#### Refinement

*R*[*F* ^2^ > 2σ(*F* ^2^)] = 0.042
*wR*(*F* ^2^) = 0.098
*S* = 1.06
3197 reflections
239 parameters
5 restraints
H atoms treated by a mixture of independent and constrained refinement
Δρ_max_ = 0.32 e Å^−3^
Δρ_min_ = −0.41 e Å^−3^
Absolute structure: Flack (1983 ▶), 1504 Friedel pairs
Flack parameter: 0.12 (8)

### 

Data collection: *COLLECT* (Hooft, 1998 ▶); cell refinement: *DENZO* (Otwinowski & Minor, 1997 ▶) and *COLLECT*; data reduction: *DENZO* and *COLLECT*; program(s) used to solve structure: *SHELXS97* (Sheldrick, 2008 ▶); program(s) used to refine structure: *SHELXL97* (Sheldrick, 2008 ▶); molecular graphics: *XP* in *SHELXTL* (Sheldrick, 2008 ▶) and *Mercury* (Macrae *et al.*, 2006 ▶); software used to prepare material for publication: *publCIF* (Westrip, 2010 ▶).

## Supplementary Material

Crystal structure: contains datablocks I, global. DOI: 10.1107/S1600536810022105/kj2150sup1.cif

Structure factors: contains datablocks I. DOI: 10.1107/S1600536810022105/kj2150Isup2.hkl

Additional supplementary materials:  crystallographic information; 3D view; checkCIF report

## Figures and Tables

**Table 1:** Hydrogen-bond geometry (Å, °)

*D*—H⋯*A*	*D*—H	H⋯*A*	*D*⋯*A*	*D*—H⋯*A*
N2—H1*N*⋯O4^i^	0.88 (2)	2.21 (4)	2.906 (4)	135 (4)
N2—H1*N*⋯O1^ii^	0.88 (2)	2.59 (4)	3.230 (4)	130 (4)
N3—H3*N*⋯O2^iii^	0.88 (2)	2.11 (3)	2.929 (5)	154 (5)
C10—H10*B*⋯O2^iv^	0.99	2.31	3.267 (5)	163

Symmetry codes: (i) 
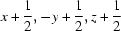
; (ii) 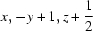; (iii) 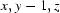; (iv) 
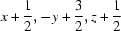
.

**Fig. 1.**
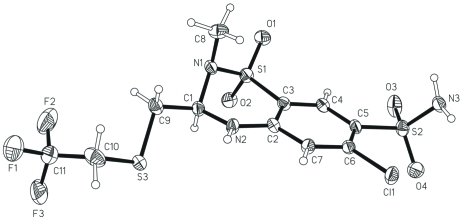
The molecular structure with displacement ellipsoids drawn at the 50% probability level. Hydrogen atoms are shown as spheres of arbitrary size.

**Fig. 2.**
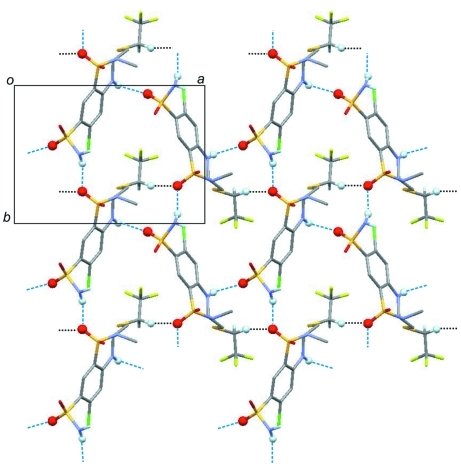
Portion of a hydrogen bonded sheet parallel to (-101) and defined by N—H···O (dashed lines) and additional C—H···O (dotted lines) contacts. H and O atoms directly involved in these interactions are drawn as balls, and hydrogen attached to C atoms are omitted for clarity.

## supplementary crystallographic information

### Comment

The title compound is a thiazide diuretic drug. The asymmetric unit contains a single molecule (see Fig. 1), and the lattice parameters are consistent with a preliminary crystallographic study (Dupont & Dideberg, 1970). The geometrical parameters of the thiazide unit are in concert with other structures of the same class of compounds (see section Related literature). The S2—N3 bond of the sulfonyl group is *gauche* with respect to the C5—C6 bond of the phenyl ring. The conformation of C—S—C—C side chain of the heterocyclic ring is characterized by the torsions angles N1—C1—C9—S3 = 170.8 (3)°, C1—C9 —S3—C10 = 162.6 (3)° and C9—S3—C10—C11 = 94.5 (4)°.

Each polythiazide molecule is N—H···O bonded to two neighbouring molecules so that a hydrogen bonded sheet parallel to (-101) is formed (see Fig. 2). The NH group and one sulfonamide O atom are engaged in an N2—H···O4(*x* + 1/2, -*y* + 1/2) interaction. An N3—H···O2(*x*, *y* - 1, *z*) bond links two molecules *via* the sulfonamide NH_2_ group and a thiazide sulfonyl O atom. Each N—H···O bonded sheet contains an additional short C10—H···O2(*x* + 1/2, -*y* + 3/2, *z* + 1/2) contact (see Table 1). A longer N2—H···O1(*x*, -*y* + 1, *z* + 1/2) contact between neighbouring sheets, in which the NH group is involved again, is also worth mentioning. The closest intermolecular contact of the second NH_2_ H atom is to the S atom of the side chain, H2N···S3(*x*, -*y* + 1, *z* - 1/2) = 2.93 (4) Å.

### Experimental

The investigated crystals were obtained from a polythiazide sample from Pfizer (Brussels, Belgium).

### Refinement

All H atoms were identified in a difference map. Methyl H atoms were idealized and included as rigid groups allowed to rotate but not tip (C—H = 0.98 Å) and refined with 1.5 *U*_eq_(C). H atoms bonded to primary (C—H = 1.00 Å), secondary CH_2_ (C—H = 0.99 Å) and aromatic carbon atoms (C—H = 0.95 Å) were positioned geometrically and refined with *U*_iso_ = 1.2 *U_eq_*(C). Hydrogen atoms attached to N were refined with restrained distances [N—H = 0.88 (2) Å]; and their *U*_iso_ parameters were refined freely.

### Crystal data

**Table:**

C_11_H_13_ClF_3_N_3_O_4_S_3_	*F*(000) = 896
*M**_r_* = 439.87	*D*_x_ = 1.700 Mg m^−^^3^
Monoclinic, *C**c*	Mo *K*α radiation, λ = 0.71073 Å
Hall symbol: C -2yc	Cell parameters from 11202 reflections
*a* = 14.6659 (7) Å	θ = 2.9–27.5°
*b* = 9.5498 (6) Å	µ = 0.64 mm^−^^1^
*c* = 13.6720 (7) Å	*T* = 120 K
β = 116.149 (3)°	Plate, colourless
*V* = 1718.87 (16) Å^3^	0.12 × 0.10 × 0.06 mm
*Z* = 4	

### Data collection

**Table:**

Bruker-Nonius Roper CCD camera on κ-goniostat diffractometer	3197 independent reflections
Radiation source: Bruker–Nonius FR591 rotating anode	2768 reflections with *I* > 2σ(*I*)
graphite	*R*_int_ = 0.055
Detector resolution: 9.091 pixels mm^-1^	θ_max_ = 26.0°, θ_min_ = 3.3°
φ & ω scans	*h* = −17→18
Absorption correction: multi-scan (*SADABS*; Sheldrick, 2007)	*k* = −11→11
*T*_min_ = 0.927, *T*_max_ = 0.963	*l* = −15→16
9021 measured reflections	

### Refinement

**Table:**

Refinement on *F*^2^	Secondary atom site location: difference Fourier map
Least-squares matrix: full	Hydrogen site location: inferred from neighbouring sites
*R*[*F*^2^ > 2σ(*F*^2^)] = 0.042	H atoms treated by a mixture of independent and constrained refinement
*wR*(*F*^2^) = 0.098	*w* = 1/[σ^2^(*F*_o_^2^) + (0.0435*P*)^2^ + 0.009*P*] where *P* = (*F*_o_^2^ + 2*F*_c_^2^)/3
*S* = 1.06	(Δ/σ)_max_ < 0.001
3197 reflections	Δρ_max_ = 0.32 e Å^−^^3^
239 parameters	Δρ_min_ = −0.41 e Å^−^^3^
5 restraints	Absolute structure: Flack (1983), 1504 Friedel pairs
Primary atom site location: structure-invariant direct methods	Flack parameter: 0.12 (8)

### Special details

**Table:**

Geometry. All e.s.d.'s (except the e.s.d. in the dihedral angle between two l.s. planes) are estimated using the full covariance matrix. The cell e.s.d.'s are taken into account individually in the estimation of e.s.d.'s in distances, angles and torsion angles; correlations between e.s.d.'s in cell parameters are only used when they are defined by crystal symmetry. An approximate (isotropic) treatment of cell e.s.d.'s is used for estimating e.s.d.'s involving l.s. planes.
Refinement. Refinement of *F*^2^ against ALL reflections. The weighted *R*-factor *wR* and goodness of fit *S* are based on *F*^2^, conventional *R*-factors *R* are based on *F*, with *F* set to zero for negative *F*^2^. The threshold expression of *F*^2^ > σ(*F*^2^) is used only for calculating *R*-factors(gt) *etc*. and is not relevant to the choice of reflections for refinement. *R*-factors based on *F*^2^ are statistically about twice as large as those based on *F*, and *R*- factors based on ALL data will be even larger.

### Fractional atomic coordinates and isotropic or equivalent isotropic displacement parameters (Å^2^)

**Table:**

	*x*	*y*	*z*	*U*_iso_*/*U*_eq_	
Cl1	0.88627 (8)	0.03391 (10)	0.90846 (8)	0.0280 (2)	
S1	0.94030 (7)	0.63949 (10)	0.76867 (7)	0.0226 (2)	
S2	0.77245 (7)	0.12532 (10)	0.65142 (8)	0.0241 (2)	
S3	1.04262 (8)	0.75479 (12)	1.17195 (8)	0.0262 (2)	
O1	0.9673 (2)	0.6291 (3)	0.6804 (2)	0.0285 (7)	
O2	0.8556 (2)	0.7293 (3)	0.7536 (2)	0.0272 (7)	
O3	0.7388 (2)	0.2033 (3)	0.5523 (2)	0.0348 (7)	
O4	0.6988 (2)	0.0628 (3)	0.6808 (2)	0.0344 (7)	
N1	1.0394 (2)	0.6927 (4)	0.8780 (3)	0.0228 (7)	
N2	1.0131 (3)	0.5322 (3)	0.9992 (3)	0.0214 (7)	
H1N	1.043 (4)	0.502 (6)	1.067 (2)	0.052 (16)*	
N3	0.8417 (3)	−0.0017 (4)	0.6449 (3)	0.0255 (8)	
H2N	0.892 (3)	0.022 (5)	0.631 (4)	0.050 (16)*	
H3N	0.856 (4)	−0.066 (4)	0.696 (3)	0.041 (15)*	
C1	1.0167 (3)	0.6786 (4)	0.9736 (3)	0.0233 (9)	
H1	0.9479	0.7195	0.9531	0.028*	
C2	0.9619 (3)	0.4380 (4)	0.9200 (3)	0.0192 (8)	
C3	0.9196 (3)	0.4722 (4)	0.8079 (3)	0.0193 (8)	
C4	0.8626 (3)	0.3757 (4)	0.7286 (3)	0.0188 (8)	
H4	0.8328	0.4027	0.6541	0.023*	
C5	0.8483 (3)	0.2403 (4)	0.7563 (3)	0.0199 (8)	
C6	0.8960 (3)	0.2032 (4)	0.8677 (3)	0.0192 (8)	
C7	0.9523 (3)	0.2976 (4)	0.9475 (3)	0.0206 (8)	
H7	0.9849	0.2686	1.0215	0.025*	
C8	1.1391 (3)	0.6397 (5)	0.8917 (3)	0.0289 (10)	
H8A	1.1933	0.6981	0.9447	0.043*	
H8B	1.1421	0.6430	0.8216	0.043*	
H8C	1.1480	0.5428	0.9180	0.043*	
C9	1.0919 (3)	0.7580 (4)	1.0712 (3)	0.0251 (9)	
H9A	1.0985	0.8557	1.0509	0.030*	
H9B	1.1595	0.7129	1.1004	0.030*	
C10	1.1529 (3)	0.8004 (6)	1.2942 (3)	0.0360 (11)	
H10A	1.1519	0.7472	1.3558	0.043*	
H10B	1.2143	0.7715	1.2865	0.043*	
C11	1.1605 (4)	0.9502 (6)	1.3201 (4)	0.0438 (13)	
F1	1.2461 (3)	0.9823 (5)	1.4089 (3)	0.0842 (14)	
F2	1.1618 (3)	1.0300 (4)	1.2398 (3)	0.0708 (10)	
F3	1.0838 (2)	0.9967 (4)	1.3395 (3)	0.0656 (10)	

### Atomic displacement parameters (Å^2^)

**Table:**

	*U*^11^	*U*^22^	*U*^33^	*U*^12^	*U*^13^	*U*^23^
Cl1	0.0427 (6)	0.0181 (5)	0.0234 (5)	−0.0054 (4)	0.0147 (4)	−0.0008 (4)
S1	0.0297 (5)	0.0179 (5)	0.0215 (5)	0.0026 (4)	0.0125 (4)	0.0038 (4)
S2	0.0276 (5)	0.0226 (6)	0.0185 (5)	−0.0012 (5)	0.0069 (4)	−0.0028 (4)
S3	0.0322 (5)	0.0254 (6)	0.0257 (5)	−0.0050 (4)	0.0172 (4)	−0.0072 (4)
O1	0.0404 (17)	0.0296 (17)	0.0201 (15)	−0.0011 (13)	0.0175 (13)	0.0038 (12)
O2	0.0278 (15)	0.0215 (16)	0.0331 (17)	0.0057 (13)	0.0141 (13)	0.0059 (13)
O3	0.0458 (18)	0.0267 (17)	0.0168 (15)	0.0028 (14)	0.0000 (12)	−0.0001 (12)
O4	0.0300 (16)	0.0406 (19)	0.0346 (18)	−0.0126 (15)	0.0161 (14)	−0.0130 (15)
N1	0.0321 (19)	0.0193 (18)	0.0214 (18)	−0.0022 (15)	0.0157 (15)	−0.0036 (14)
N2	0.0327 (18)	0.0143 (17)	0.0176 (18)	−0.0026 (14)	0.0116 (15)	0.0015 (13)
N3	0.034 (2)	0.020 (2)	0.025 (2)	0.0027 (16)	0.0156 (16)	−0.0014 (15)
C1	0.036 (2)	0.017 (2)	0.021 (2)	−0.0040 (18)	0.0165 (17)	−0.0021 (16)
C2	0.0214 (19)	0.019 (2)	0.017 (2)	0.0023 (16)	0.0083 (16)	−0.0020 (16)
C3	0.024 (2)	0.016 (2)	0.019 (2)	0.0041 (16)	0.0111 (16)	0.0020 (16)
C4	0.0179 (18)	0.019 (2)	0.017 (2)	0.0053 (16)	0.0061 (15)	0.0013 (16)
C5	0.022 (2)	0.019 (2)	0.019 (2)	−0.0006 (17)	0.0092 (17)	−0.0054 (16)
C6	0.0242 (19)	0.017 (2)	0.018 (2)	0.0004 (16)	0.0109 (16)	0.0005 (16)
C7	0.028 (2)	0.019 (2)	0.0131 (19)	−0.0031 (17)	0.0075 (15)	0.0003 (15)
C8	0.021 (2)	0.041 (3)	0.025 (2)	0.0011 (19)	0.0104 (17)	0.0052 (19)
C9	0.032 (2)	0.023 (2)	0.021 (2)	0.0007 (18)	0.0118 (17)	−0.0009 (17)
C10	0.033 (2)	0.054 (3)	0.020 (2)	0.004 (2)	0.0101 (18)	−0.002 (2)
C11	0.040 (3)	0.054 (4)	0.044 (3)	−0.020 (2)	0.024 (2)	−0.021 (3)
F1	0.066 (2)	0.128 (4)	0.062 (2)	−0.063 (2)	0.0310 (18)	−0.056 (2)
F2	0.095 (2)	0.052 (2)	0.072 (2)	−0.0414 (19)	0.043 (2)	−0.0140 (18)
F3	0.069 (2)	0.052 (2)	0.095 (3)	−0.0182 (17)	0.053 (2)	−0.0440 (19)

### Geometric parameters (Å, °)

**Table:**

Cl1—C6	1.736 (4)	C2—C3	1.415 (5)
S1—O1	1.429 (3)	C2—C7	1.416 (6)
S1—O2	1.448 (3)	C3—C4	1.388 (5)
S1—N1	1.640 (3)	C4—C5	1.390 (5)
S1—C3	1.753 (4)	C4—H4	0.9500
S2—O3	1.430 (3)	C5—C6	1.412 (5)
S2—O4	1.437 (3)	C6—C7	1.375 (5)
S2—N3	1.610 (4)	C7—H7	0.9500
S2—C5	1.759 (4)	C8—H8A	0.9800
S3—C10	1.794 (4)	C8—H8B	0.9800
S3—C9	1.816 (4)	C8—H8C	0.9800
N1—C8	1.480 (5)	C9—H9A	0.9900
N1—C1	1.490 (5)	C9—H9B	0.9900
N2—C2	1.353 (5)	C10—C11	1.467 (7)
N2—C1	1.447 (5)	C10—H10A	0.9900
N2—H1N	0.88 (2)	C10—H10B	0.9900
N3—H2N	0.87 (2)	C11—F3	1.340 (6)
N3—H3N	0.884 (19)	C11—F2	1.343 (6)
C1—C9	1.508 (5)	C11—F1	1.343 (6)
C1—H1	1.0000		
			
O1—S1—O2	117.39 (17)	C3—C4—H4	119.5
O1—S1—N1	109.12 (17)	C5—C4—H4	119.5
O2—S1—N1	107.83 (17)	C4—C5—C6	117.7 (3)
O1—S1—C3	110.15 (18)	C4—C5—S2	118.4 (3)
O2—S1—C3	109.29 (18)	C6—C5—S2	123.9 (3)
N1—S1—C3	101.91 (18)	C7—C6—C5	122.0 (4)
O3—S2—O4	119.56 (19)	C7—C6—Cl1	117.5 (3)
O3—S2—N3	107.61 (19)	C5—C6—Cl1	120.5 (3)
O4—S2—N3	105.8 (2)	C6—C7—C2	120.3 (3)
O3—S2—C5	106.11 (18)	C6—C7—H7	119.8
O4—S2—C5	108.34 (18)	C2—C7—H7	119.8
N3—S2—C5	109.11 (19)	N1—C8—H8A	109.5
C10—S3—C9	101.8 (2)	N1—C8—H8B	109.5
C8—N1—C1	116.6 (3)	H8A—C8—H8B	109.5
C8—N1—S1	116.0 (3)	N1—C8—H8C	109.5
C1—N1—S1	108.8 (2)	H8A—C8—H8C	109.5
C2—N2—C1	121.0 (3)	H8B—C8—H8C	109.5
C2—N2—H1N	118 (4)	C1—C9—S3	106.4 (3)
C1—N2—H1N	121 (4)	C1—C9—H9A	110.5
S2—N3—H2N	116 (4)	S3—C9—H9A	110.5
S2—N3—H3N	115 (3)	C1—C9—H9B	110.5
H2N—N3—H3N	115 (5)	S3—C9—H9B	110.5
N2—C1—N1	110.2 (3)	H9A—C9—H9B	108.6
N2—C1—C9	111.2 (3)	C11—C10—S3	113.7 (4)
N1—C1—C9	111.8 (3)	C11—C10—H10A	108.8
N2—C1—H1	107.8	S3—C10—H10A	108.8
N1—C1—H1	107.8	C11—C10—H10B	108.8
C9—C1—H1	107.8	S3—C10—H10B	108.8
N2—C2—C3	122.5 (4)	H10A—C10—H10B	107.7
N2—C2—C7	120.1 (3)	F3—C11—F2	106.8 (5)
C3—C2—C7	117.3 (3)	F3—C11—F1	106.1 (4)
C4—C3—C2	121.2 (4)	F2—C11—F1	105.4 (4)
C4—C3—S1	119.5 (3)	F3—C11—C10	113.0 (4)
C2—C3—S1	119.3 (3)	F2—C11—C10	112.5 (4)
C3—C4—C5	121.1 (3)	F1—C11—C10	112.5 (5)

### Hydrogen-bond geometry (Å, °)

**Table:**

*D*—H···*A*	*D*—H	H···*A*	*D*···*A*	*D*—H···*A*
N2—H1N···O4^i^	0.88 (2)	2.21 (4)	2.906 (4)	135 (4)
N2—H1N···O1^ii^	0.88 (2)	2.59 (4)	3.230 (4)	130 (4)
N3—H3N···O2^iii^	0.88 (2)	2.11 (3)	2.929 (5)	154 (5)
C10—H10B···O2^iv^	0.99	2.31	3.267 (5)	163

Symmetry codes: (i) *x*+1/2, −*y*+1/2, *z*+1/2; (ii) *x*, −*y*+1, *z*+1/2; (iii) *x*, *y*−1, *z*; (iv) *x*+1/2, −*y*+3/2, *z*+1/2.
